# Embedded Smart Antenna for Non-Destructive Testing and Evaluation (NDT&E) of Moisture Content and Deterioration in Concrete

**DOI:** 10.3390/s19030547

**Published:** 2019-01-28

**Authors:** Kah Hou Teng, Patryk Kot, Magomed Muradov, Andy Shaw, Khalid Hashim, Michaela Gkantou, Ahmed Al-Shamma’a

**Affiliations:** 1 Built Environment & Sustainable Technology (BEST) Research Institute, Faculty of Engineering & Technology, Liverpool John Moores University, Liverpool L3 3AF, UK; A.Shaw@ljmu.ac.uk (A.S.); K.S.Hashim@ljmu.ac.uk (K.H.); M.Gkantou@ljmu.ac.uk (M.G.); A.Al-Shamma’a@ljmu.ac.uk (A.A.-S.); 2 Sensor City Liverpool Limited, Liverpool L3 5LJ, UK; M.Muradov@ljmu.ac.uk

**Keywords:** concrete block, CST, deterioration, moisture content, NDT&E, smart antenna

## Abstract

Concrete failure will lead to serious safety concerns in the performance of a building structure. It is one of the biggest challenges for engineers to inspect and maintain the quality of concrete throughout the service years in order to prevent structural deterioration. To date, a lot of research is ongoing to develop different instruments to inspect concrete quality. Detection of moisture ingress is important in the structural monitoring of concrete. This paper presents a novel sensing technique using a smart antenna for the non-destructive evaluation of moisture content and deterioration inspection in concrete blocks. Two different standard concrete samples (United Kingdom and Malaysia) were investigated in this research. An electromagnetic (EM) sensor was designed and embedded inside the concrete to detect the moisture content within the structure. In addition, CST microwave studio was used to validate the theoretical model of the EM sensor against the test data. The results demonstrated that the EM sensor at 2.45 GHz is capable of detecting the moisture content in the concrete with linear regression of R^2^ = 0.9752. Furthermore, identification of different mix ratios of concrete were successfully demonstrated in this paper. In conclusion, the EM sensor is capable of detecting moisture content non-destructively and could be a potential technique for maintenance and quality control of the building performance.

## 1. Introduction

Concrete structures play an important role in structural health of buildings, bridges, and so forth. [1]. It is normal that concrete undergoes deterioration over time due to the natural behaviour of the concrete [2]. However, it undergoes rapid deterioration when exposed to aggressive environments and unpredictable weather changes, which leads to a reduction in service life. Hence, it is important to ensure the service life of concrete structures are monitored [3]. Premature deterioration of the structure has a huge cost related impact on national economics. About 3% of the world’s gross domestic product (GDP) that is, US$ 2.2 trillion lost has been reported from world statistics owing to a premature deterioration of concrete structures [4]. In addition, structural health plays a significant role for the risk of building collapse, which may cause injuries or even death to occupants [5]. Hence, reliability of concrete and reinforced concrete (RC) structures can be ensured through accurate prediction of service life, which is influenced by several deteriorating factors such as corrosion of embedded metals, chemical attack, erosion, surface defect and so forth [6].

Extensive research studies have reported that moisture content within the concrete structures is a key point for structural health monitoring [7,8]. The moisture induces varying forms of physical, biological and chemical processes, which rapidly deteriorate the structural strength [9]. Currently, there are a few conventional methods available to determine the dampness of building fabrics [10]. However, most of the available equipment to measure moisture content is destructive to the building fabric, as they require additional drilling into the material to take a measurement. Moreover, these methods often provide inaccurate results [11]. Hence, it creates a huge research interest to develop non-destructive methods and reliable solutions for structural health monitoring [12]. Throughout the years, many non-destructive techniques have been reported in the literature to monitor the moisture content in concrete, such as thermal analysis [13], gamma ray method [14], the ultrasonic techniques [15], X-ray diffraction [16] and scanning electron microscopy [17]. Reported results showed that these methods provided much better performance and accuracy compared to the conventional methods. However, precise calibration, high skills for operating and use of these techniques is required [18]. Therefore, it is necessary to develop easy, reliable and user-friendly technology/instruments to monitor the structural health of buildings, bridges and so forth.

In recent years, numerous studies specialized in the structural health monitoring (SHM) investigating new smart material-based methods to replace the conventional techniques [19]. These smart material-based SHM techniques include piezoelectric materials [20] and fibre optic sensors [21,22] to monitor damage, debonding and corrosion. However, monitoring of moisture content which is the key factor for the structural life prediction has been disregarded [23]. Recently, a few research activities introduced an electro-mechanical impedance (EMI) technique to monitor the concrete hydration, curing and strength gain [24]. Qin and Li monitored the hydration of cement using embedded piezoelectric patch (PZT), by determining the dynamic modulus of the cement paste through the measurement of the ultrasonic pulse velocity. However, they could not monitor the early hydration as they bonded the PZT patch to the concrete surface only after hardening [25]. Providakis et al. designed a miniaturized wireless EMI based measuring system to monitor the early age strength of concrete. Based on their experimental studies, they found that the EMI signal gradually shifted to the right as the concrete curing time increased [26]. Different sensing techniques with polymer optic fibres have been proposed throughout the year [27,28]. Zhou et al. introduced fibre-reinforced polymer-packaged optical-fibre Bragg grating (OFBG) to monitor damage for civil infrastructure under harsh environment [29]. Furthermore, there are different patch antennas were proposed recently for varies application for structure heat monitoring. Jun Yao et al. demonstrated that wireless vibratory strain sensing system for dynamic strain tracking ability based on principle of resonant frequency as a function of the tensile strain [30]. On the other hand, Sanders et al. found that microstrip patch antenna is a potential to use as temperature sensor by correlating the antenna resonant frequency shift and the temperature change [31]. On top of that, Cheng et al. also has demonstrated the microstrip patch antenna for high temperature sensor application [32].

Hence, this research paper aims to develop a smart sensing technology by using electromagnetic wave techniques for the determination of moisture content and therefore deterioration in concrete structures. A microstrip patch antenna developed to generate EM signals and continuous data monitoring was performed by transmitting information to the data acquisition system. The smart sensor is embedded inside the concrete structure for real-time monitoring. A model for determination of moisture content and deterioration of concrete is successfully developed and implemented. Furthermore, theoretical model of signal transmitting of electromagnetic wave was validated by using CST microwave simulation software. This smart sensor can be easily applied to determine moisture content of concrete in real time and user-friendly approach.

## 2. Experimental Setup and Methodology

### 2.1. Microwave Theory and Application

Moisture content inside concrete structures can be studied from the data of their interactions with microwaves. This interaction can be revealed in the form of a unique signal spectrum called as reflection coefficient (S_11_) and transmission coefficient (S_21_) [33]. Permittivity and conductivity of water percentages will vary the measurement quantities of signal spectrum. Permittivity is a measurement of the dielectric medium response to the applied microwaves through the changing of its electric field. It depends on the material’s ability to polarize in response to the applied field. This theory can be applied to detect moisture in concrete structures because water generally has high value of dielectric constant ~81 [34]. Dielectric constant and dielectric loss of material is the two key parameters to define permittivity.

- Dielectric constant (ε′) is defined as a quantity measuring the ability of a material to store electrical energy in an electric field. The changing moisture content of concrete will vary the dielectric constant due to the polarization of water inside the concrete sample.
- Dielectric loss (ε″) is defined as loss of electromagnetic energy propagating inside the concrete structure due to the rotation and oscillation between the water molecules thus resulting in friction.

Changes of moisture content inside the concrete structure will alter its permittivity and yielding a unique spectrum when it comes in contact with microwave [35]. Hence, this technology is suitable and fits well to evaluate the moisture content inside the concrete [36].

### 2.2. Microstrip Patch Antenna at 2.45 GHz Frequency

**Figure 1:**
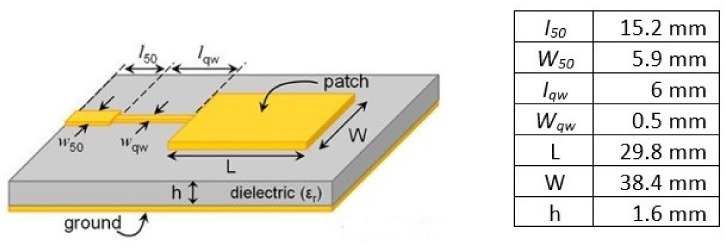
Full dimension of a patch antenna for 2.45 GHz frequency.

CST Microwave Studio was used to design a patch type sensor that resonates at 2.45 GHz frequency for this investigation. The designed sensor was proposed under consideration of the desired resonant frequency, dielectric medium of the patch antenna and size consideration for overall rectangular patch antenna. Figure 1 illustrates the full dimension of a patch antenna propagated at 2.45 GHz frequency. A microstrip patch antenna consists of a conducting patch (copper) and ground plane in between (FR-4). FR-4 is a dielectric medium known as substrate. Table 1 shows the properties of the materials and the overall dimension that includes material thickness, dielectric constant, electrical conductivity and loss tangent of the material.

#### Design of Microstrip Patch Antenna

The width (W) of the patch is calculated by Equation (1):

(1) $$W=\frac{c}{2f_{o}}\sqrt{\frac{2}{\varepsilon_{r}+1}}$$

where c is free space speed of light, $f_{o}$ is desired resonant frequency and $\varepsilon_{r}$ is dielectric constant of substrate.

The length (L) of the patch is calculated by Equation (2):

(2) $$L=\;\frac{c}{2f_{o}\sqrt{\varepsilon_{eff}}}-0.824h\left(\frac{\left(\varepsilon_{eff}+0.3\right)\left(\frac{W}{h}\;+\;0.264\right)}{\left(\varepsilon_{eff\;}-\;0.258\right)\left(\frac{W}{h}\;+\;0.8\right)}\right),\;\varepsilon_{eff}=\frac{\varepsilon_{r}\;+\;1}{2}+\frac{\varepsilon_{r\;}-\;1}{2}\left(\frac{1}{\sqrt{1\;+\;12\frac{h}{W}}}\right).$$

where $\varepsilon_{eff}$ is the effective dielectric constant, W is width of the patch and h is height of substrate.

A small square size of 60 mm × 60 mm ground was incorporated in the patch antenna. The substrate size has the similar size to the ground. Up to date research, there are different methods of feeding to the microstrip patch antenna such as inset feed method, fed with a quarter-wavelength transmission line, coaxial cable, coupled feeds and aperture feeds. In this research, we aim to produce a remarkable small size, simple fabrication and easy integration antenna. With the benefits of minimal dependence on substrate thickness and unipolar configuration, quarter-wavelength transmission feed line was selected for the patch antenna.

### 2.3. Materials Preparation and Properties

#### 2.3.1. Fabrication of Printed Circuit Board (Patch Antenna)

**Figure 2:**
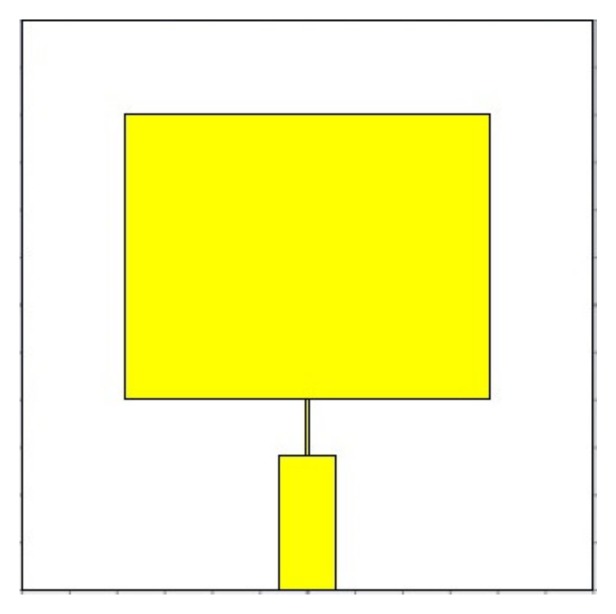
Designed 2.45 GHz Microstrip Patch Antenna Sensor ready for fabrication.

Once the sensor is designed and a desired performance is obtained, the next step was to fabricate it. EAGLE (Easily Applicable Graphical Layout Editor) was used prior to moving to the actual fabrication of the antenna. EAGLE is a PCB (Printed Circuit Board) design software, which is developed by CadSoft (CadSoft US, 2016). Figure 2 shows the circuit layout ready to print on the PCB for the patch antenna sensor. The software contains a number of functions, such as a schematics editor, a PCB editor and auto-router module. The most common use of this software is a design of electronic schematics and layouts of PCB boards.

**Figure 3:**
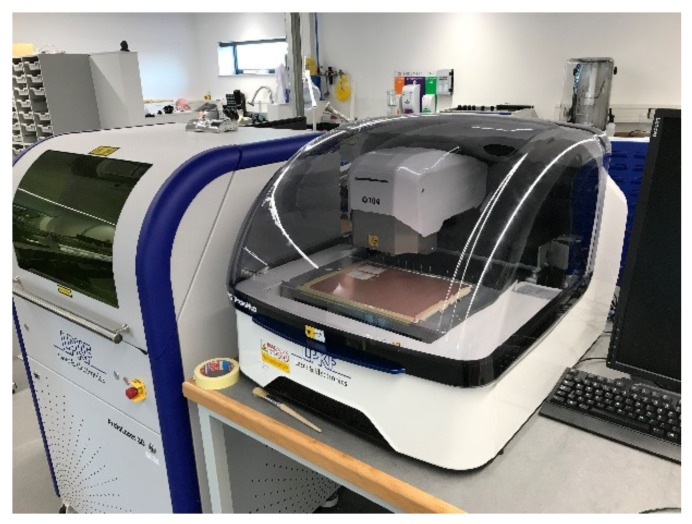
LPKF Protomat D104 machine used to fabricate the microstrip antenna on FR4 PCB board.

Figure 3 shows the LPKF Protomat D104, which was used to fabricate the antenna on FR4 PCB board. The machine was controlled by Circuit Pro control software via a PC, where the antenna’s layout files were loaded for drilling and the milling. The Gerber files were exported from Autodesk EAGLE software.

#### 2.3.2. Concrete Structure

A preliminary cubes structure for testing was made from two different standards (Europe EN 206-1-2013 and Malaysia MS 26-1-8:2010). The size of the block is 150 × 300 × 300 mm there were no reinforcement bars. The composition of the concrete structures is provided in Table 2.

#### 2.3.3. Water Content

An electronic weight balance was placed under the concrete block throughout the experiments. The weight before and after the test are recorded to measure the weight of moisture content in the concrete block. Equation (3) was used to evaluate the water content measurement throughout the experiments.

(3) $$\mathrm{MC}=W_{w}-W_{d}$$

were MC is moisture content weight (kg), $W_{w}$ is wet weight (kg) and $W_{d}$ is dry weight (kg) of overall concrete.

### 2.4. Experimental Setup

**Figure 4:**
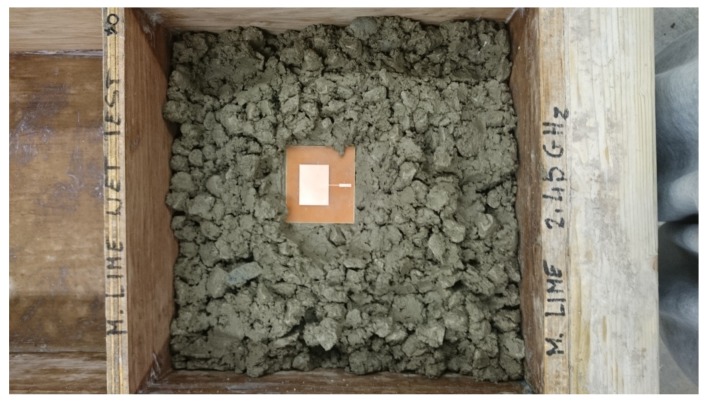
Smart sensor is embedded in the concrete block for moisture detection.

A printed circuit board (smart sensor) transmitted signal of 2.45 GHz was designed at Liverpool John Moores University and fabricated at Sensor City Liverpool Limited. The smart sensor is embedded within the concrete structure as shown in Figure 4.

**Figure 5:**
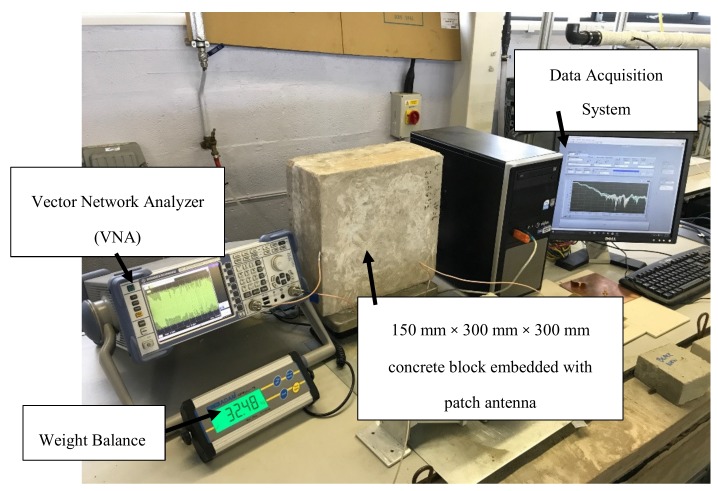
Experimental Setup for moisture content detection in concrete structure.

The experimental setup for this investigation is shown in Figure 5. The experimental setup consists of a vector network analyser (VNA), weight balance under the concrete and a data acquisition system designed in LabVIEW program. Reflection signals (S_11_) were detected from the patch antenna connected to the VNA. All the results were recorded throughout the experiments to quantify the moisture content lost.

### 2.5. Data Acquisition

A continuous monitoring was implemented using VNA for attenuation of the electromagnetic wave propagation in the concrete and weight of water content by electronic weight balance. All the experimental results are recorded by using designed LabVIEW Program in the interval of 5 min for 48 h. Three repeatability test for each of the samples were performed. Root means square (RMS) error was calculated and was found to be >95% for the repetition.

### 2.6. Numerical Simulation

Numerical simulation was conducted by using CST simulation package in order to stimulate the resonant frequency for the microstrip patch antenna. The simulation results were set at range of 1.8 GHz–3.0 GHz and were validated against the experimental results.

## 3. Results and Discussions

### 3.1. Moisture Content in MS 26-1-8:2010 Concrete

**Figure 6:**
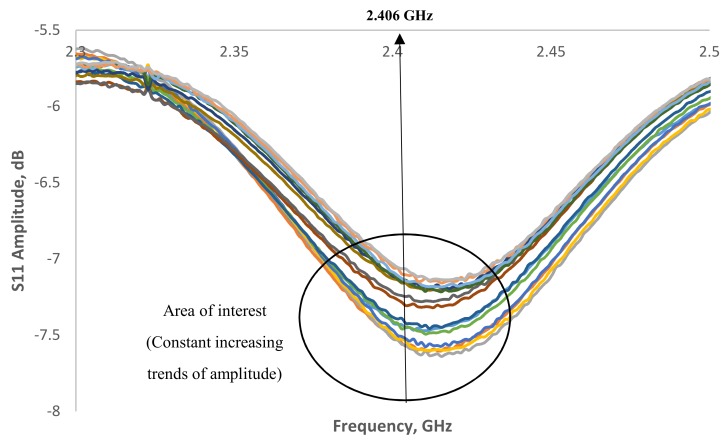
Signal spectrum for moisture content in MS 26-1-8:2010 concrete structure in the range of 2.3–2.5 GHz.

**Figure 7:**
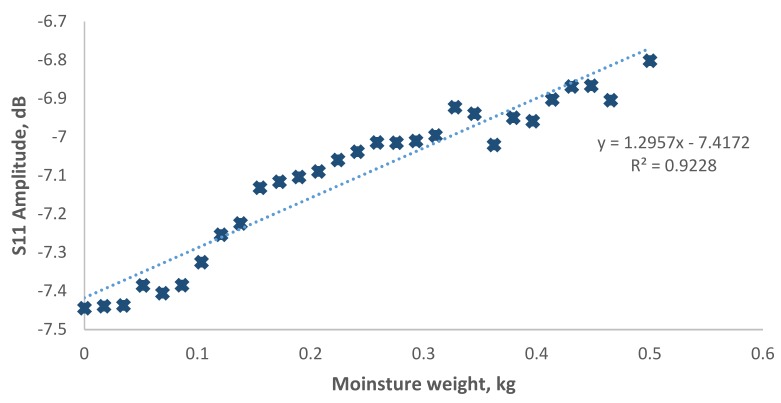
Correlation of moisture content weight with S11 amplitude at 2.406 GHz in MS 26-1-8:2010 concrete.

Signal spectrum generated from the smart sensor inside the MS 26-1-8:2010 concrete is illustrated in Figure 6. It is interesting to see that at the frequency of 2.4 GHz, the signal spectrum from −7.5 dB to −6.8 dB when the moisture content decrease. Figure 7 shows the correlation of frequency shift against the moisture content inside the concrete. A linear regression of 0.9228 which suggesting that this signal region could be potential for moisture content measurements.

### 3.2. Moisture Content in Europe EN 206-1-2013 Concrete in 2.43 GHz and 4.16 GHz

**Figure 8:**
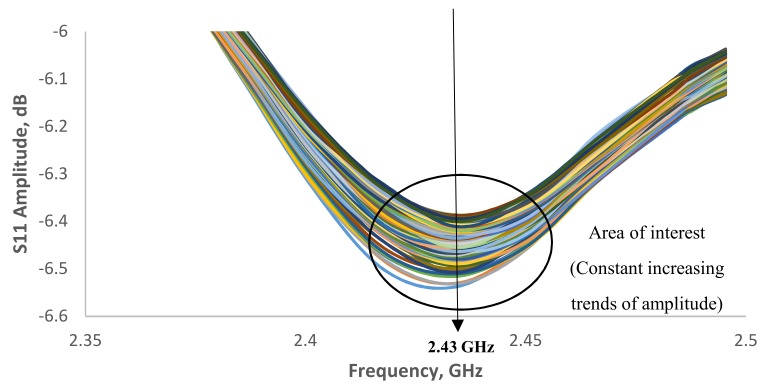
Signal spectrum for moisture content in EN 206-1-2013 concrete structure in the range of 2.35–2.5 GHz.

**Figure 9:**
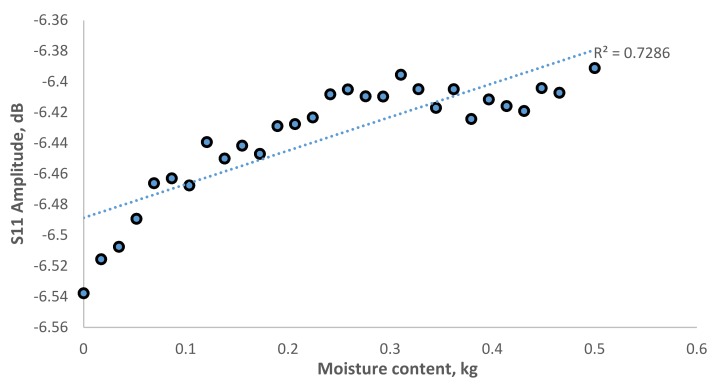
Correlation of S11 amplitude against moisture content in Europe EN 206-1-2013 correlation in 2.43 GHz.

**Figure 10:**
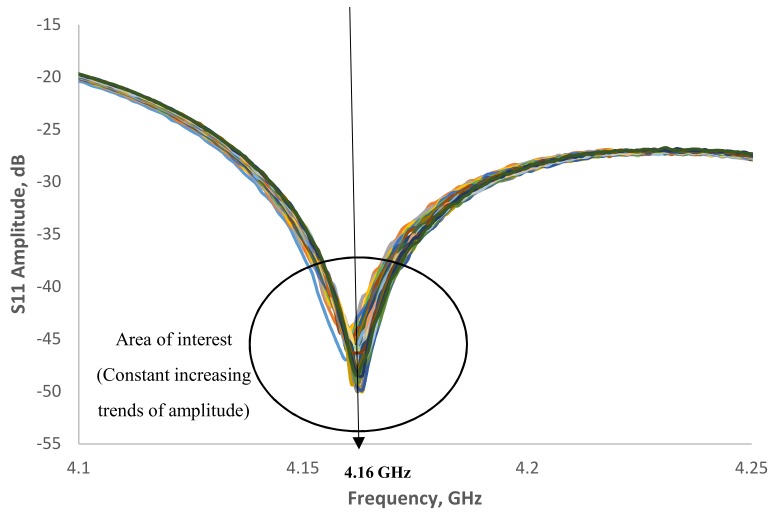
Signal spectrum for moisture content in EN 206-1-2013 concrete structure in the range of 4.1–4.25 GHz.

**Figure 11:**
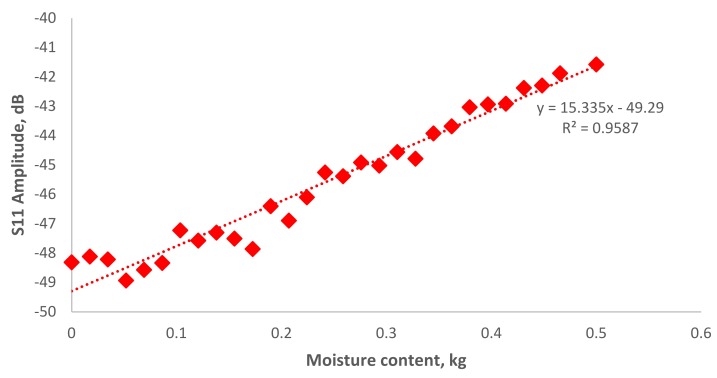
Correlation of S11 amplitude against moisture content in Europe EN 206-1-2013 correlation in 4.16 GHz frequency.

Figure 8 shows the signal spectrum for moisture content in EN 206-1-2013 concrete in a range of 2.35–2.5 GHz reported a small fraction of amplitude increase from −6.5 to −6.39 dB. Figure 9 show the correlation of amplitude with moisture content at R^2^ = 0.7286. The results show a fair correlation in the region of 2.43 GHz. Further investigation was carried on for the area of interest around 4.16 GHz, which is the second harmonic wave of the designed 2.45 GHz antenna sensor as show in Figure 10. The amplitude increases from −48.3 to −41.5 dB when moisture content decrease. In Figure 11, it is shown the correlation between the amplitude and moisture content. A better linear regression of moisture content relationship with signal amplitude of 4.16 GHz at R^2^ = 0.9587 can be observed compare to 2.43 GHz. Hence, it is concluded that the designed microstrip patch antenna can accurately monitor the moisture content of the concrete and hence deterioration monitoring of concrete structures.

### 3.3. Signal Spectrum for MS 26-1-8:2010 Concrete and Europe EN 206-1-2013 Concrete 

**Figure 12:**
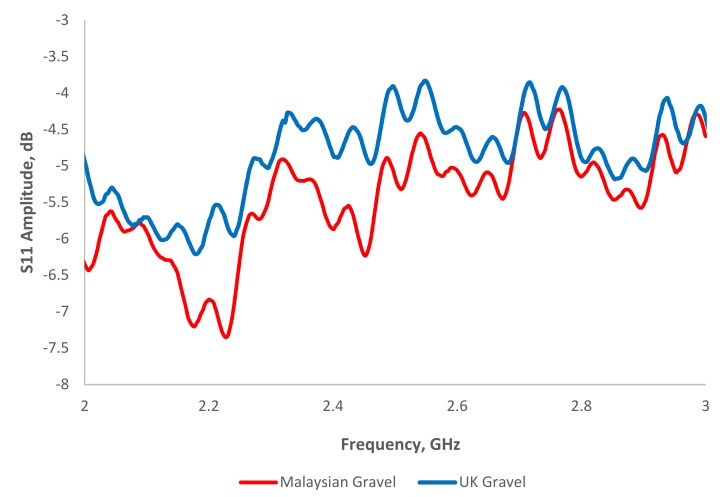
Signal spectrum for MS 26-MS 26-1-8:2010 concrete and Europe EN 206-1-2013 concrete in the range of 2–3 GHz at dry condition.

Figure 12 illustrates the signal spectrum of a smart antenna embedded inside two different concrete blocks. Generally, two-signal spectrums show a similar pattern trend but different amplitude values in the range of 2–3 GHz. The difference in the reflected signal variation can be related to the variation in the concrete’s composition. Future work could be interesting to investigate the patch antenna for composition detection for quality issue approach.

### 3.4. Simulation Results

**Figure 13:**
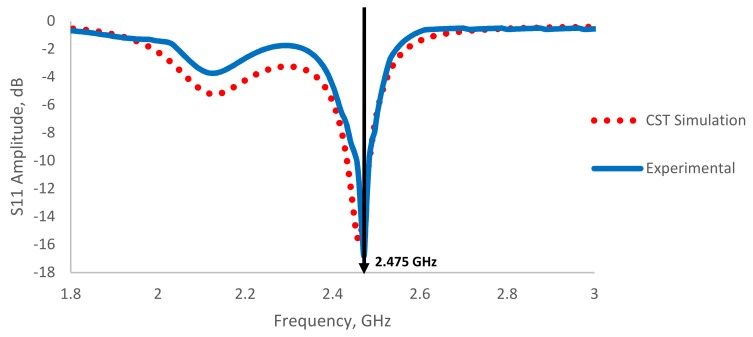
Comparison between experimental and CST simulation results of patch antenna in exposure to air condition.

Figure 13 shows the simulated results by using CST studio suit compared to experimental results. It is shown that the signal spectrum is well simulated with 95% of root mean square error. The simulation is well designed for the amplitude peak at 2.45 GHz at −15.6 dB. However, the experimental results show the peak at 2.475 GHz at 16.8 dB, which offset 0.02 GHz from the simulation design. The variation is due to the accuracy of the actual patch antenna dimension and thickness of the materials. Future work is recommended for the high precision machining for production of patch antenna.

## 4. Conclusions

The EM sensor showed high potential to be used for moisture loss evaluation in concrete structures. The following conclusions can be drawn:

(1) Designed of 2.45 GHz patch antenna is well validated between experiment and simulation.
(2) Self-printed 2.45 GHz patch antenna successfully detected moisture content in two concrete blocks that were made based on two different standards (Europe EN 206-1-2013 and Malaysia MS 26-1-8:2010).
(3) Detection of moisture content in Malaysia MS 26-1-8:2010 and Europe EN 206-1-2013 concrete blocks is well correlated at linear regression of 0.9228 and 0.9587 respectively.

The future investigation is to implement the full sensing system, namely circuitries of RF signal generator inside the concrete by self-generated energy for data transmission for moisture detection.

## Figures and Tables

**Table 1:** Properties of conductor and dielectric of microstrip patch antenna.

	Copper (Conductor & Ground)	FR-4 (Dielectric)
Thickness	0.03 mm	1.6 mm
Dielectric constant, ε	1	4.7
Electrical Conductivity	5.96 × 10^7^ (S/m)	0
Loss tangent, tan *б*	0	0.035

**Table 2:** Concrete composition with OPC (CEM II 32.5 R)- kg/m^3^.

Parameters	UK Standards (EN 206-1-2013)	Malaysian Standards (MS 26-1-8:2010)
Type of cement	CEM II	CEM II
Cement	350	393
Sand 0.25–2 mm	525	589
Gravel 4–8 mm	1050	1178
Water/cement ratio	0.7	0.5
